# Periprosthetic bone loss after insertion of an uncemented, customized femoral stem and an uncemented anatomical stem: A randomized DXA study with 5-year follow-up

**DOI:** 10.3109/17453674.2011.588860

**Published:** 2011-09-02

**Authors:** Mona Nysted, Pål Benum, Jomar Klaksvik, Olav Foss, Arild Aamodt

**Affiliations:** ^1^Department of Orthopaedic Surgery, Trondheim University Hospital; ^2^Department of Neuroscience, Norwegian University of Science and Technology, Trondheim, Norway; Correspondence: mona.nysted@ntnu.no

## Abstract

**Background and purpose:**

Customized femoral stems are designed to have a perfect fit and fill in the femur in order to achieve physiological load transfer and minimize stress shielding. Dual-energy X-ray absorptiometry (DXA) is regarded as an accurate method for detection of small alterations in bone mineral density (BMD) around hip prostheses. We present medium-term DXA results from a randomized study comparing a customized and an anatomical femoral stem.

**Methods:**

100 hips were randomized to receive either the anatomical ABG-I stem or the Unique customized femoral stem, both uncemented. DXA measurements were conducted postoperatively and after 3, 6, 12, 24, 36, and 60 months, and BMD was computed for each of the 7 Gruen zones in the proximal femur.

**Results:**

Results from 87 patients were available for analysis. 78 completed the 5-year follow-up: 35 patients in the ABG group and 43 patients in the Unique group. In both groups, we found the greatest degree of bone loss in the proximal Gruen zones. In zone 1, there was 15% reduction in BMD in the ABG-I group and 14% reduction in the Unique group. In zone 7, the reduction was 28% in the ABG-I group and 27% in the Unique group. The only statistically significant difference between the groups was found in Gruen zone 4, which is distal to the tip of the stem, with 1.6% reduction in BMD in the ABG-I group and 9.7% reduction in the Unique group (p = 0.003).

**Interpretation:**

5-year DXA results showed that because of stress-shielding, proximal bone loss could not be avoided—either for the anatomical ABG-I stem or for the customized Unique stem.

Implantation of a prosthesis in the femur alters the load distribution in the host bone, and the femur remodels to adapt to the new mechanical situation (Engh and Bobyn 1988, Bobyn et al. 1992, Engh et al. 1999). A perfect fit and fill in the proximal femur is said to be important to achieve physiological load transfer (Laine et al. 2000). It has therefore been hypothesized that a customized femoral stem could fulfill the criterion for optimal fit and fill, and thus minimize stress shielding in the proximal femur (Bargar 1989). Based on numerical analyses, however, it has been claimed that canal-filling femoral stems may cause stress shielding and subsequently bone atrophy of the proximal femur (Huiskes et al. 1989).

In vitro studies on human cadaver femurs have indicated that insertion of a customized stem gives a better pattern and distribution of cortical strains in the proximal femur than anatomical stems (Aamodt et al. 2001, Østbyhaug et al. 2009). In these studies, it was shown that 33–56% of the external cortical strains were retained on the proximal, medial aspect of the femur after insertion of a customized stem whereas the corresponding figure was 10–13% after insertion of a standard, anatomical stem. Long-term bone remodeling is, however, not necessarily reflected in the immediate postoperative mechanical situation in the femur as measured in experimental strain analyses.

The present randomized, clinical study was undertaken to compare (by DXA) the medium-term changes in bone mineral density in the proximal femur after insertion of an uncemented, customized femoral stem and an uncemented, standard anatomical femoral stem.

## Patients and methods

Patients less than 65 years of age with primary osteoarthritis or secondary osteoarthritis due to hip dysplasia, Legg-Calve-Perthes disease, trauma, or avascular femoral head necrosis were eligible for inclusion. Patients with abnormal size and geometry of the proximal femur, who were considered unsuitable for a standard femoral prosthesis, were excluded. All patients signed an informed consent form and the study was approved by the Regional Ethics Committee (No. 70-98). The study was conducted according to the requirements of the Helsinki Declaration.

**Figure 1.**
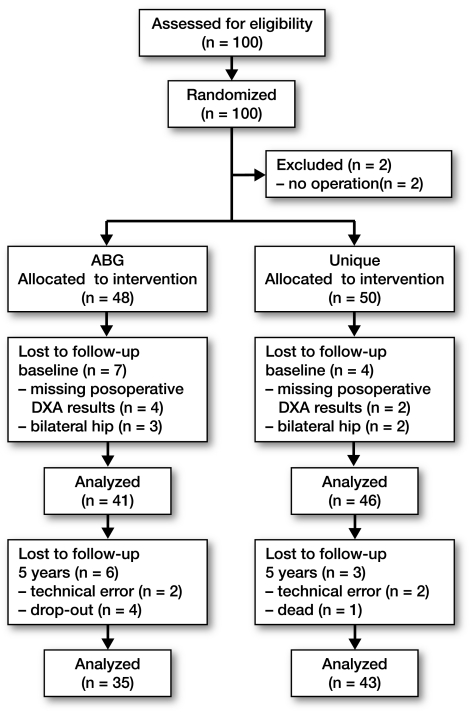
Consort flow chart.

We enrolled 100 hips in the study (Figure 1). The inclusions were done between January 1999 and April 2001. Non-computerized randomization, in blocks of 10, was carried out. The code was kept in a sealed, opaque envelope and it was broken at the outpatient clinic after the patients had signed the informed consent form. 50 hips were scheduled to receive a Unique uncemented, customized femoral stem (SCP, Trondheim, Norway) and 50 hips were scheduled to receive the uncemented, anatomical ABG-I (Stryker-Howmedica, Allendale, NJ). Due to a manufacturing time of 4–6 weeks for the customized prostheses, randomization took place during a preoperative outpatient visit and 2 patients randomized to the ABG-I group withdrew from the study due to delay of the operation. 6 patients were excluded due to inadequate postoperative DXA measurements. 5 patients with bilateral hip replacements were originally included. Later, the first hip to be operated in each of these patients was excluded from the study to maintain independence of data. Results from 87 patients were available for analysis; 78 of these patients completed the 5-year follow-up—35 patients in the ABG-I group and 43 patients in the Unique group.

31 men and 56 women were included; the mean age was 53 (21–65) years in the ABG-I group and 55 (36–65) years in the Unique group (Table 1). The diagnoses were primary osteoarthritis (49), dysplasia of the hip (29), Legg-Calve-Perthes disease (4), posttraumatic osteoarthritis (3), and avascular necrosis (2).

**Table 1.** Study samples

	ABG-I femoral stem	Unique femoral stem
No. of patients	41	46
No. of females / males	28 / 13	28 / 18
Primary ostearthrtitis	49%	63%
Hip dysplasia	44%	24%
Sequelae: Perthes' disease	2%	7%
Posttraumatic	2%	4%
Avascular femoral head necrosis	2%	2%

After 5 years, no hips had been revised but 5 patients reported complications. In the ABG-I group 2 patients had an early dislocation, 1 patient developed a deep venous thrombosis, and 1 had an episode of subluxation. In the Unique group, 1 patient suffered from an intraoperative injury of the common peroneal nerve.

### Implants

**Figure 2.**
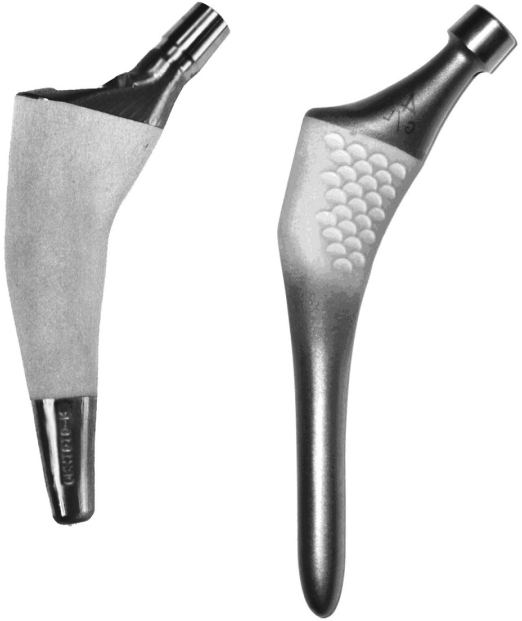
The implants. The Unique stem (left) and the ABG-I (right).

Both stems are made of titanium alloy (Ti_6_Al_4_Va) with hydroxyapatite coating (HA), and they have a modular femoral head and no collar (Figure 2). The design of the Unique customized stem is based on 2-D, cross-sectional CT scans of the proximal femur. Using an interactive design algorithm, closed contours were generated along the corticocancellous interface of the femoral canal. It has been shown that a CT density of 600 Hounsfield Units (HU) represents this interface (Aamodt et al. 1999). The stems were designed to fit closely to the inner cortical surface in the metaphyseal region in order to obtain maximum mechanical stability and optimal load transfer. The stem has a circumferential plasma-sprayed HA coating of 50 µm thickness and 62% crystallinity on its proximal two-thirds. The distal third is unpolished and has a roughness of 2.5 µm. It is downscaled to prevent distal locking and load transfer. A resection guide mounted on an intramedullary reamer was used to achieve the preplanned resection level on the femoral neck. The femoral canal was prepared using a custom-made broach that had the same dimensions as those of the prosthesis, except that its diameter was 1 mm larger distal to the coating area. No diaphyseal reaming was performed.

The ABG-I stem is an anatomical stem with a press-fit metaphyseal fixation. At the metaphyseal level, the prosthesis is covered with a plasma-sprayed HA layer with a thickness of 60 µm and a crystallographic composition of 98–99% (Giannikas et al. 2002). The coating area has a macro relief scaled surface designed to transform shear forces into compression forces (Tonino et al. 1995). The distal portion of the implant is designed to avoid endomedullary contact with the diaphysis (Panisello et al. 2009b). Before implantation of the ABG-I stem, the medullar canal was prepared with 1 mm over-reaming, and then sequential rasping of the metaphysis was performed.

Stem size and neck resection was decided by preoperative and intraoperative planning. On the acetabular side, we used an uncemented Duraloc component (DePuy, Leeds, UK), except for 2 cases, which were operated with autologous impaction bone grafting and a cemented Elite plus Ogee cup (DePuy). Both patients were in the Unique group. Postoperatively, the patients were allowed full weight bearing with 2 crutches for two months. Antibiotic prophylaxis was given for the first 24 h and low-molecular-weight heparin for the first 14 days.

4 experienced orthopedic surgeons performed all the procedures and a standard direct lateral approach was used. The patients were clinically assessed using the Merle d'Aubigne (MdA) score for measurement of pain, joint mobility, and ability to walk (d'Aubigne and Postel 1954).

### Bone densitometry

**Figure 3.**
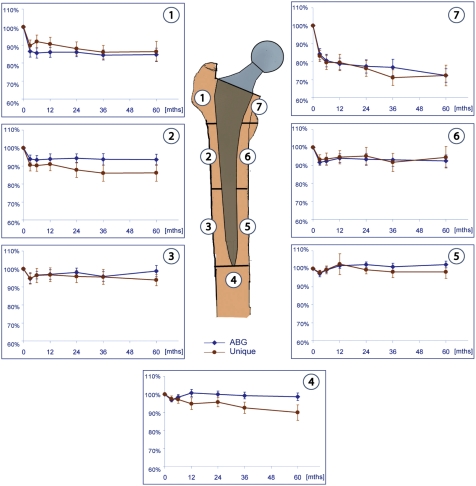
Average bone mineral density (%, with 95% CI) in the 7 Gruen zones from baseline to 5-year follow-up.

To evaluate changes in bone mass, the patients were examined using a Hologic QDR 4500 DXA scanner (Hologic Inc., Bedford, MA) within the first week and after 3, 6, 12, 24, 36, and 60 months. Scanning was performed with the patient in supine position. The leg was placed in a standardized support to ensure a neutral position. Bone mineral density (BMD) in the frontal plane of the femur was measured according to the 7 Gruen zones. Figure 3 shows the anatomical landmarks that divide the zones; in the horizontal plane, the tip of the lesser trochanter defines the distal border of zones 1 and 7. The midpoint between the lesser trochanter and the tip of the stem defines the border between zones 2 and 3, and 5 and 6. Zone 4 represents the total bone area 20 mm distally from the tip of the stem. Vertically, the center axis of the femur divides the medial and lateral zones. Postoperative measurements were used as baseline values.

### Statistics

Our main aim was to evaluate changes in bone density around 2 uncemented hip arthroplasties and to compare the groups after 5 years. The BMD measurements were normally distributed. Comparison of data between the groups was carried out using an independent-samples Student t-test procedure. Baseline results were compared to subsequent measurements with a paired Student's t-test. Postoperative measurements were used as baseline values and the measurements at follow-up were expressed as a percentage of the baseline measurements. Due to multiple comparisons, the significance level was adjusted to p ≤ 0.007 according to the Bonferroni correction.

## Results

The mean relative changes in BMD (Tables 2 and 3), for each of the 7 Gruen zones from baseline to 5 years, were plotted separately for both arthroplasties (Figure 3). There was no significant difference in baseline global BMD between the groups (p = 0.9). After 5 years, we measured a global BMD loss of 7.7% in the ABG-I group and 11% in the Unique group (p = 0.09). When we compared baseline DXA values in each Gruen zone, we found significant changes in zones 1, 6, and 7 (p < 0.005) between the stems. In the ABG-I group, we noted a reduction in BMD in all Gruen zones except zone 5. There were significant changes in zones 1, 2, 6, and 7 (p < 0.001) when comparing the changes in BMD from baseline to 5-year follow-up. Bone loss was greatest in proximal zones 1 and 7. In Gruen zone 1, in the ABG-I group, there was a 14% reduction in BMD after 2 years and a further decrease to 15% after 5 years. The greatest decline in BMD was found in Gruen zone 7. After 2 years there was a 22% reduction in BMD, and a further loss up to 28% after 5 years.

**Table 2.** Mean (SD) values of BMD (in g/cm^2^) in the 7 Gruen zones from baseline to 5 years in the ABG-I group

Gruen zone	Baseline	3 months	6 months	12 months	24 months	36 months	60 months
1	0.78 (0.1)	0.68 (0.1)	0.67 (0.13)	0.67 (0.13)	0.67 (0.14)	0.67 (0.15)	0.66 (0.15)
2	1.52 (0.26)	1.44 (0.29)	1.42 (0.28)	1.42 (0.29)	1.43 (0.27)	1.43 (0.24)	1.40 (0.26)
3	1.64 (0.26)	1.55 (0.24)	1.58 (0.21)	1.58 (0.19)	1.59 (0.21)	1.58 (0.19)	1.60 (0.22)
4	1.73 (0.22)	1.68 (0.23)	1.70 (0.22)	1.74 (0.22)	1.73 (0.22)	1.73 (0.22)	1.71 (0.24)
5	1.63 (0.23)	1.58 (0.26)	1.61 (0.25)	1.66 (0.23)	1.66 (0.23)	1.66 (0.23)	1.65 (0.24)
6	1.43 (0.27)	1.31 (0.26)	1.34 (0.25)	1.34 (0.22)	1.33 (0.24)	1.32 (0.23)	1.32 (0.27)
7	1.22 (0.28)	1.03 (0.25)	1.01 (0.26)	0.97 (0.25)	0.95 (0.25)	0.93 (0.24)	0.89 (0.26)
Global	1.18 (0.19)	1.09 (0.20)	1.09 (0.17)	1.10 (0.17)	1.10 (0.17)	1.10 (0.17)	1.09 (0.18)

**Table 3.** Mean (SD) values of BMD (in g/cm^2^) in the 7 Gruen zones from baseline to 5 years in the Unique femoral group

Gruen zone	Baseline	3 months	6 months	12 months	24 months	36 months	60 months
1	0.70 (0.11)	0.63 (0.13)	0.64 (0.14)	0.63 (0.14)	0.62 (0.15)	0.59 (0.14)	0.61 (0.19)
2	1.42 (0.27)	1.30 (0.33)	1.29 (0.32)	1.30 (0.36)	1.26 (0.36)	1.21 (0.34)	1.28 (0.32)
3	1.71 (0.26)	1.63 (0.33)	1.65 (0.37)	1.64 (0.32)	1.63 (0.30)	1.61 (0.35)	1.62 (0.27)
4	1.72 (0.21)	1.69 (0.23)	1.67 (0.24)	1.65 (0.25)	1.66 (0.27)	1.59 (0.29)	1.57 (0.31)
5	1.71 (0.20)	1.67 (0.18)	1.69 (0.19)	1.69 (0.20)	1.69 (0.20)	1.66 (0.21)	1.68 (0.24)
6	1.13 (0.25)	1.06 (0.27)	1.06 (0.27)	1.07 (0.27)	1.08 (0.28)	1.03 (0.29)	1.10 (0.34)
7	1.01 (0.21)	0.84 (0.23)	0.81 (0.23)	0.80 (0.24)	0.77 (0.25)	0.72 (0.23)	0.75 (0.27)
Global	1.15 (0.15)	1.07 (0.17)	1.06 (0.17)	1.03 (0.22)	1.04 (0.18)	1.00 (0.17)	1.04 (0.20)

The trend was similar in the Unique group, where we found significant differences in BMD in Gruen zones 1, 2, 3, 4, and 7 after 5 years compared to baseline measurements (p < 0.001). In zone 1, there was 13% loss after 2 years—increasing to 14% after 5 years. In zone 7, the decrease was 24% after 2 years and 28% after 5 years. In the middle areas (in zones 2, 3, 5, and 6) there were only small changes in BMD from 2 to 5 years in both groups. Implantation of a femoral stem affected the BMD in zone 5 to a small extent. There was full recovery in bone density around both stems after 1 year, and the bone mineral density was unaltered after 5 years. In the distal area (zone 4), we found a significant difference between the groups at 5 years. There was only a slight reduction in bone mineral density of 1.6% in the ABG-I group as compared to 9.9% reduction in BMD in the Unique group (p = 0.003). In all other zones, we found no statistically significant differences in BMD between the two groups.

The clinical scores were similar between the groups, both preoperatively and at 5 years. The mean preoperative Merle-d'Aubigne score was 11 (7–14) in the ABG-I group and 10 (6–13) in the Unique group. The mean scores after 5 years were 17 in both groups.

## Discussion

Dual-energy X-ray absorptiometry (DXA) is regarded as an accurate method for detection of small changes in bone mineral density (BMD) close to hip arthroplasties (Kiratli et al. 1992, Cohen and Rushton 1995, Kiratli et al. 1996). In this study, DXA measurements showed a difference in baseline results following insertion of the anatomical ABG-I stem and the customized Unique stem, when data were analyzed according to the 7 Gruen zones. We found higher postoperative BMD values in proximal zones 1, 6, and 7 of femurs in patients with the ABG-I stem than in the corresponding zones of femurs in patients with the Unique stem. The explanation for this difference may be found in the way the femurs were prepared before inserting the stems. The design of the custom stem implies that more bone would be removed from the metaphysis during rasping than the amount of bone removed during stepwise rasping with the ABG-I broaches. We also observed that there was a higher proportion of secondary hip arthrosis in the Unique group, compared to the ABG-I group (Table 1), but we did not find any difference in postoperative BMD when comparing the subgroups of patients according to diagnosis (p > 0.17). We therefore believe that the difference in baseline results in the proximal zones may be attributed to the different ways of preparing and rasping the proximal femur. We found a pronounced decline in BMD in the proximal Gruen zones both in the ABG-I group and the Unique group after 2 years; thereafter, there was a tendency of a small shift in bone mass between zones from 2 to 5 years. These findings correspond to reports from other authors reporting on bone loss and remodeling after insertion of cementless THRs (Wixson et al. 1997, Rosenthall et al. 1999, Tanzer et al. 2001, Venesmaa et al. 2001, Grant et al. 2005, Panisello et al. 2009a). There were no significant differences between the 2 stems regarding bone loss in the proximal Gruen zones at 5 years.

In Gruen zone 4, on the other hand, the ABG arthroplasty gave better bone preservation than the Unique stem. The ABG-I stem has a grit-blasted surface texture distally, while the Unique stem is downscaled distally to prevent distal locking. The grit-blasted texture of the ABG-I might favor distal bone preservation (van der Wal et al. 2008).

To prevent bone resorption in the upper femur after implantation of a femoral stem, the prevailing theory is to have a physiological transfer of loads from the prosthetic head to cortical bone in the metaphysis. The Unique stem has an optimal fit to the inner cortical surface in the metaphyseal region, and was designed to load the proximal part of the femur in order to minimize stress shielding (Aamodt et al. 2001). In fact, in vitro analysis of strain on human cadaver femurs has shown that cortical strains in the proximal femur are retained after insertion of a custom femoral stem to a significantly higher degree than after insertion of a standard, anatomical stem (Aamodt et al. 2001, Østbyhaug et al. 2009).

The Unique stem has an HA coating extending approximately 15–20 mm below the tip of the lesser trochanter, which may encourage fixation in the distal part of the coated area and hence stress shielding in the metaphyseal area (Sumner and Galante 1992). This may explain the lower magnitude of bone loss in Gruen zones 3 and 5 than in the proximal lateral and medial zones. After 5 years, there was bone loss of 1.2% in zone 3 and a gain of 1.9% in zone 5, which may indicate distal fixation and stress relief in zones 1 and 7. Although it has not been shown experimentally or clinically, it is possible that the extent of the HA-coating, and thus the area of secondary fixation, is more important for the pattern of load transfer than the fit and fill of the stem. The limited metaphyseal bone loss measured after implantation of ultra-short femoral stems supports this hypothesis (Albanese et al. 2009). In order to maintain strains in the metaphyseal part of the femur, after biological fixation, a stem with a high degree of metaphyseal fit and fill should probably not be designed to obtain biological fixation below the lesser trochanter.

Due to the canal-filling design feature of the Unique stem, the implant has a large cross-sectional geometry, which makes it relatively stiff. Size and stiffness of the femoral implant are considered to be important factors regarding bone remodeling around hip arthroplasties, and negative bone remodeling is more apparent around larger and stiffer stems (Engh and Bobyn 1988, Huiskes et al. 1992, Sumner and Galante 1992, Wixson et al. 1997, Skoldenberg et al. 2006). Reduced bone loss has been demonstrated around isoelastic, flexible stems as compared to titanium alloy arthroplasties (Ang et al. 1997, Karrholm et al. 2002). Both the ABG-I stem and the Unique stem have gone through some modifications since our study was conducted. The second generations of both arthroplasties have smaller metaphyseal volume for similar sizes. Other modifications for ABG-II include the use of less stiff titanium alloy and polishing of the distal part of the diaphyseal stem (Van der Wal et al. 2006, 2008, Herrera et al. 2009). The Unique stem has had a proximal porous coating added under the HA layer, and the stem distal to the coated area is now highly polished (Benum and Aamodt 2010).

Grant et al. (2005) compared the Unique stem with a cemented arthroplasty and published 2-year DXA results. They reported a greater reduction in BMD around the Unique stem compared to the results in our study. In zone 1, we found a 13% reduction in BMD after 2 years, as opposed to the 22% reduction reported in the study by Grant et al. There was an even more pronounced decline in zone 7, with a bone loss of 24% and 32%, respectively. The results of Grant et al. and those in our study revealed a higher degree of proximal bone loss than in previous investigations on custom-made implants (Martini et al. 1996, Zerahn et al. 1998, Leichtle et al. 2006). These authors reported a reduction in proximal BMD of between 10% and 15% at the end of the third year. Grant et al. had a smaller study population: 38 patients as compared to 83 in our study. Neither Grant et al. nor we measured BMD preoperatively, which Rahmy et al. (2004) stated as an important factor for prediction of bone loss postoperatively. Since we had almost the same distribution regarding age and gender of the populations, we do not believe that the two populations would have differed in preoperative bone mass.

The bone loss observed did not appear to affect the clinical outcome of our patients, which was similar to that in previous reports on these stems (Rogers et al. 2003, Herrera et al. 2004, Rahmy et al. (2004, Benum and Aamodt 2010).

As far as we know, no other randomized studies have led to 5-year DXA results comparing different designs of cementless femoral stems. Our study has some limitations. No power analysis was performed before the study started. The sample size estimate was based on studies in the literature. Rahmy et al. (2004) and van der Wal et al. 2008) stated that the preoperative BMD was the most important factor for prediction of bone loss following a hip arthroplasty. They recommended that the patients should be matched for preoperative bone quality in randomized studies. In this study, we did not obtain DXA measurements of the hip or other skeletal regions before the hip arthroplasty. Instead, we used the immediate postoperative BMD measurements as baseline values for computation of the subsequent changes in periprosthetic bone mass. In addition, due to bone resorption, the proximal femur in zone 7 changes its contour line and often diminishes. This may make the DXA measurement in this zone less accurate.

The medium- and long-term clinical consequences of the proximal bone loss around uncemented stems are uncertain. No aseptic loosening has been observed radiologically in large series of hips followed prospectively for 7 and 10 years after implantation of the Unique stem (Benum and Aamodt 2010). The implants used in our study were both stiff, canal-filling stems, which may have contributed to negative bone remodeling. The extent of the HA layer and the unpolished surface of the distal part of the Unique stem may have encouraged distal fixation and bone loss in the proximal region. Bone remodeling after total hip arthroplasty is affected by several different factors, and together with the stress-shielding, bone loss around a femoral stem may also occur as a result of an inflammatory reaction to wear particles (Schmalzried and Callaghan 1999, Rahmy et al. (2004). Our study illustrates that the predictive value of in vitro tests regarding strain distribution should not be overestimated.
